# Physical Activity Increases White Matter Microstructure in Children

**DOI:** 10.3389/fnins.2018.00950

**Published:** 2018-12-19

**Authors:** Laura Chaddock-Heyman, Kirk I. Erickson, Caitlin Kienzler, Eric S. Drollette, Lauren B. Raine, Shih-Chun Kao, Jeanine Bensken, Robert Weisshappel, Darla M. Castelli, Charles H. Hillman, Arthur F. Kramer

**Affiliations:** ^1^Beckman Institute, University of Illinois at Urbana-Champaign, Urbana, IL, United States; ^2^Department of Psychology, University of Pittsburgh, Pittsburgh, PA, United States; ^3^Department of Psychology, University of Colorado, Denver, CO, United States; ^4^Department of Kinesiology, University of North Carolina at Greensboro, Greensboro, NC, United States; ^5^Department of Psychology, Northeastern University, Boston, MA, United States; ^6^Department of Kinesiology and Community Health, University of Illinois at Urbana-Champaign, Urbana, IL, United States; ^7^Department of Kinesiology and Health Education, The University of Texas at Austin, Austin, TX, United States; ^8^Department of Physical Therapy, Movement, & Rehabilitation Sciences, Northeastern University, Boston, MA, United States

**Keywords:** brain deve, children, physical activity, diffusion tensor imaging, white matter

## Abstract

Children are becoming increasingly inactive, unfit, and overweight, yet there is relatively little causal evidence regarding the effects of physical activity on brain health during childhood. The present study examined the effects of an after-school physical activity program (FITKids2) on the microstructure of white matter tracts in 7- to 9-year-old children. We measured the microstructural properties of white matter via diffusion tensor imaging in 143 children before and after random assignment to either a 9-month after-school physical activity program (*N* = 76, mean age = 8.7 years) or a wait list control group (*N* = 67, mean age = 8.7 years). Our results demonstrate that children who participated in the physical activity program showed increased white matter microstructure in the genu of the corpus callosum, with no changes in white matter microstructure in the wait list control group which reflects typical development. Specifically, children in the physical activity program showed increases in fractional anisotropy (FA) and decreases in radial diffusivity (RD) in the genu from pre- to post-test, thereby suggesting more tightly bundled and structurally compact fibers (FA) and increased myelination (RD), with no changes in estimates of axonal fiber diameter (axial diffusivity, AD). The corpus callosum integrates cognitive, motor, and sensory information between the left and right hemispheres of the brain, and the white matter tract plays a role in cognition and behavior. Our findings reinforce the importance of physical activity for brain health during child development.

## Introduction

Children are becoming increasingly inactive, unfit, and overweight. Exercise has decreased in school-aged youth, with only one-quarter of children participating in the recommended 60 min or more of moderate-to-vigorous physical activity per day (National Physical Activity Plan Alliance, 2016). Schools, which reach ~55.5 million children between the ages of 5 and 17 years (National Center for Education Statistics, U.S. Department of Education, 2017), have contributed to the declining health of youth through the implementation of policies aimed at minimizing physical activity opportunities during the school day in an effort to improve academic performance (Institute of Medicine, 2013). However, such policies are not supported by empirical evidence. In fact, a growing number of studies demonstrate that an active and fit lifestyle is beneficial for cognitive and brain health across the lifespan (see Chaddock et al., 2012; Kramer and Colcombe, 2018 for reviews). Participation in physical activity and higher levels of aerobic fitness are positively related to scholastic performance, cognitive function, and brain health. Specifically, physically active and higher fit children outperform less active and lower fit children in and out of the classroom, and these performance differences are paralleled by differences in the structure and function of the brain (see Chaddock-Heyman et al., 2014b for review and Donnelly et al., 2016 for review).

To date, most research using magnetic resonance imaging (MRI) in children offers correlational evidence, with significant associations among aerobic fitness, cognition, and brain structure and function (Chaddock et al., 2010a,b; Chaddock-Heyman et al., 2013, 2014a, 2015; Esteban-Cornejo et al., 2014; Ortega et al., 2017). There is less causal evidence showing that participation in physical activity modifies the brain, with only a small number of randomized controlled trials that include neuroimaging methods (Davis et al., 2011; Kamijo et al., 2011; Chaddock-Heyman et al., 2013; Hillman et al., 2014; Krafft et al., 2014; Drollette et al., 2018). That is, few studies have manipulated physical activity behaviors to investigate whether physical activity improves brain health at a critical period of development. Consequently, the effects of physical activity on brain development are not well-understood. This is an important limitation given that children are becoming increasingly sedentary and overweight, and the developmental years are among the most sensitive periods for brain growth and development (Giedd et al., 1999; Casey et al., 2008).

The present study examined the effects of an after-school physical activity program on the microstructure of white matter tracts in children. Maturation of white matter tracts is an important element of development, as microstructural integrity of white matter is required for efficient transmission of information between gray matter as well as the integration of brain areas into structural networks to support cognitive function (Schmithorst and Yuan, 2010). During childhood and pre-adolescence, many white matter tracts throughout the brain increase in estimates of microstructure, in parallel with improvements in cognition (Barnea-Goraly et al., 2005; Muetzel et al., 2008; see Schmithorst and Yuan, 2010, for a review). Diffusion tensor imaging (DTI) allows scientists to indirectly quantify the microstructural components of white matter, including myelination and axonal organization. In general, developmental studies using diffusion weighted imaging techniques demonstrate age-related increases in fractional anisotropy (FA) coupled with decreases in diffusivity (radial diffusivity [RD] and axial diffusivity [AD]) in most white matter regions (Lebel et al., 2008; Schmithorst and Yuan, 2010; Peters et al., 2012; Tamnes et al., 2012), thereby suggesting increased axon caliber and myelin content as well as changes in fiber packing density with development (Beaulieu, 2002; Paus, 2010; Simmonds et al., 2014). Specifically, FA is a general index of white matter microstructure, hypothesized to be higher in tightly bundled, structurally compact fibers with high integrity (Basser, 1995; Beaulieu, 2002; Sen and Basser, 2005; Rykhlevskaia et al., 2008). RD is often used as a marker of myelination (Song et al., 2002, 2003, 2005; Nair et al., 2005; Budde et al., 2007; Rykhlevskaia et al., 2008), and AD is known to be sensitive to changes in axonal fibers including axonal diameter, loss or damage (Song et al., 2003; Budde et al., 2007). By exploring effects of physical activity on multiple measures of diffusivity (FA, RD, AD), we can investigate microstructural white matter properties influenced by physical activity during child development.

A few studies to date suggest that aerobic fitness and participation in physical activity play a role in white matter microstructure during childhood (Chaddock-Heyman et al., 2014a; Krafft et al., 2014; Schaeffer et al., 2014). For example, higher aerobic fitness levels relate to greater FA in 9- and 10-year old children in a diffuse set of tracts (Chaddock-Heyman et al., 2014a) including the corpus callosum, corona radiata, and superior longitudinal fasciculus. The associations with FA were primarily characterized by differences in RD (and not AD), raising the possibility that estimates of myelination may vary as a function of individual differences in fitness during childhood. In addition, two studies from a randomized controlled trial explored the effects of an after-school aerobic exercise program (40 min/day for 8 months; instructor-led aerobic activities, e.g., tag and jump rope) on white matter microstructure of two fiber tracts in a small sample of unfit, overweight 8- to 11-year-old children (*N* = 10 Exercise, *N* = 8 Control, BMI>85th percentile, 94% African American) (Krafft et al., 2014; Schaeffer et al., 2014). Neither study demonstrated Group × Time interactions for white matter structure. However, one of the studies suggested that greater attendance in the aerobic exercise program was associated with increased estimates of white matter microstructure (FA, RD) in a frontal-parietal white matter tract, the superior longitudinal fasciculus, with no relationship observed in the inactive control group that participated in art and board games (Krafft et al., 2014). The second study from the same randomized controlled trial demonstrated that the exercise group showed greater change in the microstructure (FA, RD) of a frontal-temporal white matter tract, the uncinate fasciculus (Schaeffer et al., 2014), compared to the change scores of the control group. Clearly, although these three studies provide a basis for associations among aerobic fitness, physical activity, and white matter microstructure, additional research is needed with larger and more diverse samples.

Here, we go beyond prior reports by investigating the effects of a 9-month randomized controlled physical activity trial on white matter microstructure in tracts throughout the brain during child development. We measured aerobic fitness (VO_2max_) and the microstructural properties (FA, RD, AD) of white matter in 7–9-year-old children before and after randomization into a 9-month physical activity intervention or a wait list control group (which reflects typical development over that period of time). By examining the effects of physical activity on multiple measures of diffusivity (FA, RD, AD), we were able to test the influence of physical activity on specific microstructural white matter properties during childhood. In particular, we examined the effects of physical activity on the microstructure of the following tracts identified in previous work (Chaddock-Heyman et al., 2014a; Krafft et al., 2014; Schaeffer et al., 2014): the corpus callosum which connects the left and right cerebral hemispheres and facilitates interhemispheric communication and the exchange of cognitive, motor, and sensory integration between the hemispheres, the corona radiata with ascending and descending fibers from the cerebral cortex, the superior longitudinal fasciculus, which provides bidirectional information transfer between the frontal and parietal cortex (Krafft et al., 2014), the posterior thalamic radiation, which connects the thalamus and caudate nucleus with the cerebral cortex, and the uncinate fasciculus which connects frontal and temporal regions. We hypothesized that, relative to the control condition, the FITKids2 physical activity intervention would result in increases in FA and reductions in RD estimates of white matter microstructure, with no changes in AD.

## Methods

### Participants

Children were recruited from schools in East-Central Illinois. Eligible participants were required to (1) be 7- to 9-years-old, (2) have an absence of school-related learning disabilities (i.e., individual education plan related to learning), adverse health conditions, physical incapacities, or neurological disorders, (3) qualify as prepubescent (Tanner pubertal timing score ≤ 2) (Taylor et al., 2001), (4) report no use of medications that influence central nervous system function, (5) demonstrate right handedness (as measured by the Edinburgh Handedness Questionnaire) (Oldfield, 1971), (6) successfully complete a mock MRI session to screen for claustrophobia, and (7) sign an informed assent approved by the Institutional Review Board of the University of Illinois at Urbana-Champaign. A legal guardian also provided written informed consent in accordance with the Institutional Review Board of the University of Illinois at Urbana-Champaign. The guardian was asked to provide information regarding participants' socioeconomic status (SES), as determined by: (1) participation in free or reduced-price lunch program at school, (2) the highest level of education obtained by the mother and father, and (3) number of parents who worked full-time (Birnbaum et al., 2002). Participants also completed the Woodcock Johnson III paper and pencil task of General Intellectual Ability to obtain an intelligence quotient (IQ) (Woodcock, 1997).

### Protocol

All children were asked to complete demographic assessments, a VO_2max_ test to assess aerobic fitness, and a magnetic resonance imaging (MRI) session, which included a DTI scan at pre-test (before randomization of group assignment) and post-test (after the completion of the intervention, ~9 months later). Randomization was performed by a staff member who was not involved in data collection, and group allocation was concealed from the research team until the completion of the trial. Blinding of staff and students involved in data collection and analysis was successful as there were no reported breaches of blinding throughout the course of the study. The study was carried out in accordance with the recommendations of the Institutional Review Board of the University of Illinois at Urbana-Champaign, and our protocol was approved by the Institutional Review Board.

### Intention-to-Treat

**Figure 1:**
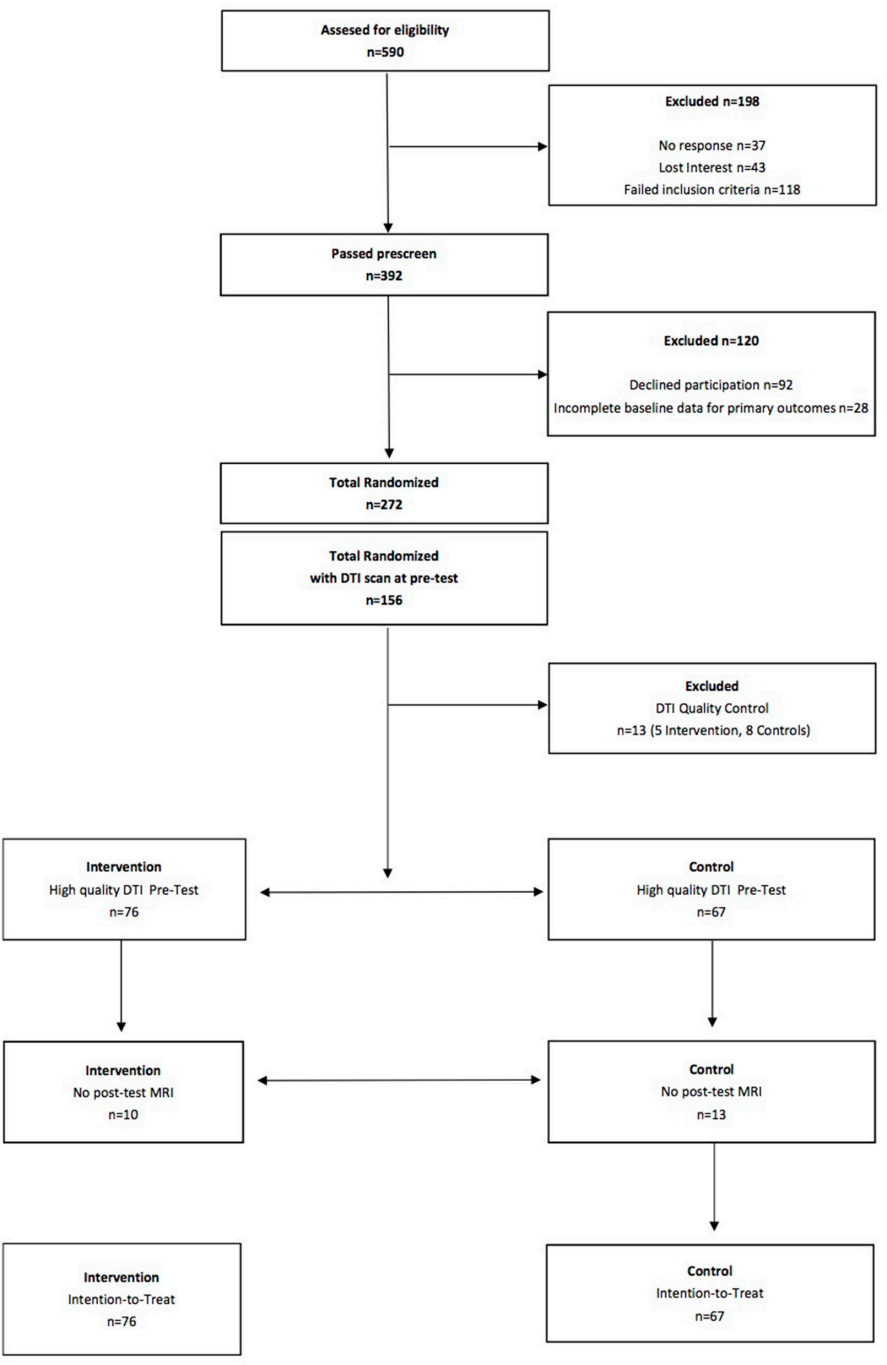
Flow diagram of the FITKids2 DTI participants.

See Figure 1 for a flow diagram of the FITKids2 DTI participants. Intention-to-treat analyses were performed for all children who completed the pre-test DTI assessment (*N* = 156) and were randomly assigned. Missing data at post-test were resolved via imputation with mean replacement (*N* = 23; 10 in the physical activity condition, 13 in the wait list control condition). Thirteen children (5 in the physical activity condition, 8 in the wait list control condition) were excluded from analysis due to visible motion on the reconstructed DTI data and/or lack of whole-brain coverage during acquisition. The intention-to-treat sample consisted of 143 children (*N* = 76 in the physical activity condition, 67 in the wait list control condition). We also conducted a sensitivity analysis by performing the analysis on only the children who completed both the pre-test DTI assessment and post-test DTI assessment (*N* = 119; 66 in the physical activity condition, 54 in the wait list control condition). The results were identical, so we only report the results from the intention-to-treat approach.

### Aerobic Fitness Testing

Children completed a VO_2max_ test to assess aerobic fitness. The aerobic fitness of each child was measured as maximal oxygen consumption (VO_2max_) during a graded exercise test (GXT). The GXT employed a modified Balke Protocol and was administered on a LifeFitness 92T motor-driven treadmill (LifeFitness, Schiller Park, IL) with expired gases analyzed using a TrueOne2400 Metabolic Measurement System (ParvoMedics, Sandy, Utah). Children walked and/or ran on a treadmill at a constant speed with increasing grade increments of 2.5% every 2 min until volitional exhaustion occurred.

Oxygen consumption was measured using a computerized indirect calorimetry system (ParvoMedics True Max 2400) with averages for VO_2_ and respiratory exchange ratio (RER) assessed every 20 s. A polar heart rate (HR) monitor (Polar WearLink+ 31; Polar Electro, Finland) was used to measure HR throughout the test, and ratings of perceived exertion (RPE) were assessed every 2 min using the children's OMNI scale (Utter et al., 2002). Maximal oxygen consumption was expressed in mL/kg/min and VO_2max_ was based upon maximal effort as evidenced by (1) a plateau in oxygen consumption corresponding to an increase of < 2 mL/kg/min despite an increase in workload; (2) a peak HR ≥ 185 beats per minute (American College of Sports Medicine, 2006) and a HR plateau (Freedson and Goodman, 1993); (3) RER ≥ 1.0 (Bar-Or, 1983); and/or (4) a score on the children's OMNI ratings of perceived exertion (RPE) scale ≥ 8 (Utter et al., 2002).

### Physical Activity Training Intervention and Wait List Control Group

The physical activity intervention occurred for 2 h after each school day from September until May for 150 days of the 170-day school year. The program, Fitness Improves Thinking in Kids 2 (FITKids2) (https://clinicaltrials.gov/ct2/show/NCT01619826?term=hillman&age=0&fund=0&rank=1) (NICHD grant HD069381, ClinicalTrials.gov, Identifier: NCT01619826) was based on the Child and Adolescent Trial for Cardiovascular Health (CATCH) curriculum (McKenzie et al., 1994) and aimed at improving aerobic fitness through engagement in a variety of developmentally appropriate physical activities. The environment was non-competitive and integrated activities such as fitness activities, motor skill practice, and low organized games similar to tag (Castelli et al., 2011).

Within a daily lesson, children participated in moderate to vigorous physical activity (recorded by E600 Polar heart rate monitors; Polar Electro, Finland, and Accusplit Eagle 170 pedometers, San Jose CA) for 30–35 sustained minutes and then intermittently up to 90 min, thus exceeding the national physical activity guideline of 60 min of moderate to vigorous physical activity per day (Centers for Disease Control and Prevention, 2012). Overall, children spent ~50% during the intervention engaged in moderate to vigorous physical activity (i.e., > 70% of heart rate max, based on pre-test VO_2_ maximal heart rate).

Each lesson began with the children completing stations that focused on a specific health-related fitness component (e.g., cardiorespiratory endurance, muscular strength). The activities were aerobically demanding and designed to encourage children to improve on previous performances by gradually increasing the number of repetitions or amount of resistance at a station. Although the stations were organized by health-related fitness components, each activity also required a motor or manipulative skill (e.g., dribbling a basketball around cones for 30-s, performing a sit-up, throwing a ball over head). After the sustained participation and active rest rotations, the children consumed a healthy snack and were introduced to a themed educational component related to health promotion (e.g., goal setting, self-management). Each lesson concluded with the children participating in non-elimination, small group games and activities such as dance or sport activities with modified rules selected from the CATCH curriculum. On the weekends, the children were encouraged to continue their participation in physical activity with their families, and physical activity worksheets were utilized during school holidays to log continued engagement. Average attendance across the 9-month intervention was 84.16% (*SD* = 12.28%).

The wait list control group completed all facets of the pre-test and post-test similar to those children who were randomized into the after-school physical activity program. As incentive to stay in the study, children in the wait list control group were afforded the opportunity to participate in the physical activity program during the following school year.

### MRI Acquisition

Diffusion weighted images were acquired using a single-shot diffusion-weighted EPI sequence in each research participant with the following parameters: TR = 7,300 ms; TE = 97 ms; FOV = 240 mm; slice thickness = 2.0 mm; acquisition matrix = 128 × 128). Thirty diffusion weighted images were acquired along 30 non-collinear directions with a b-value of 1,000 s/mm2 along with 2 images with b = 0 s/mm2. The scan time was 4 min and 17 s.

Diffusion information can be represented mathematically as a diffusion tensor / diffusion ellipsoid. FA is calculated from the three eigenvalues (λ1, λ2, λ3) of the diffusion tensor and represents anisotropic (directionally dependent) diffusion (Basser, 1995; Beaulieu, 2002; Sen and Basser, 2005), independently of the rate of diffusion. FA ranges from 0 to 1, with higher values reflecting increased directionality of diffusion (i.e., water traveling more parallel to a tract compared to perpendicularly). In a region with free diffusion, the FA value is 0 and the diffusion is isotropic. If the diffusion is more in one direction, i.e., anisotropic diffusion, the FA value approaches 1. FA is a general index of white matter microstructure, hypothesized to be higher in tightly bundled, structurally compact fibers with high integrity (Basser, 1995; Beaulieu, 2002; Sen and Basser, 2005; Rykhlevskaia et al., 2008).

We also explored specific patterns of diffusivity [radial diffusivity (RD) and axial diffusivity (AD)], hypothesized to reflect potential biological properties of white matter microstructure (Basser, 1995; Pierpaoli and Basser, 1996; Pierpaoli et al., 1996; Song et al., 2002). For example, a reduction in RD is observed in the presence of remyelination, causing RD to be often used as a marker of myelination (Song et al., 2002, 2003, 2005; Nair et al., 2005; Budde et al., 2007; Rykhlevskaia et al., 2008). RD is the average of the second and third eigenvectors (λ2, λ3), reflective of diffusivity perpendicular to the major axis of the tensor. RD reflects the rate of radial diffusion, with lower values reflecting less diffusion, and thus, increased estimates of myelination (Basser, 1995; Pierpaoli and Basser, 1996; Pierpaoli et al., 1996; Song et al., 2002).

AD is the diffusion along the principal diffusion eigenvalue (λ1) of the ellipsoid. AD is said to be sensitive to changes in axonal fibers including axonal diameter, loss or damage (Song et al., 2003; Budde et al., 2007).

### Diffusion Data Analysis

Image analyses and tensor calculations were performed using FSL 5.0.1 (FMRIB Software Library). First, each participant's data were passed through a pipeline consisting of (1) motion and eddy current correction, (2) removal of non-brain tissue using the Brain Extraction Tool (Smith, 2002), and (3) local fitting of the diffusion tensor model at each voxel using FMRIB's Diffusion Toolbox v2.0 (FDT: http://www.fmrib.ox.ac.uk/fsl/fdt). The products of the multi-step pipeline included FA and AD images. RD maps were calculated as the mean of the second and third eigenvectors (Song et al., 2002).

Next, diffusion data were processed using TBSS v1.2 (Tract-Based Spatial Statistics, Smith et al., 2006). Each participant's FA data were aligned into the 1 × 1 × 1 mm^3^ standard Montreal Neurological Institute (MNI152) space via the FMRIB58_FA template using the FMRIB's Non-linear Registration Tool (Andersson et al., 2007a,b), and a mean diffusion image was created. The mean FA image was then thinned to create an average skeleton representing the centers of the tracts shared by all participants, and the skeleton was thresholded at FA>0.20. Each participant's aligned FA data were projected onto the skeleton, taking on the FA value from the local center of the nearest relevant tract. RD and AD skeletons for each participant were formed in a similar manner by projecting the analogous data onto the mean skeleton.

### Region-of-Interest Analysis

Diffusion values (FA, RD, AD) were calculated for each participant within bilateral *a priori* regions of interest (ROIs), created from the JHU ICBM-DTI-81 white matter labels atlas (http://www.fmrib.ox.ac.uk/fsl/data/atlas-descriptions.html#wm [Mori et al., 2005; Wakana et al., 2007; Hua et al., 2008]). Tract ROIs were created in the corpus callosum, corona radiata, superior longitudinal fasciculus, posterior thalamic radiation, and uncinate fasciculus. An FSL command, fslmaths, was used to create each ROI (e.g., fslmaths JHUAtlas –uthr –thr). An average diffusion value across left and right hemispheres was computed for each ROI for each diffusion measure for each participant.

### Statistical Analysis

Analyses were conducted via a 2 (group: intervention, wait list control) × 2 (time: pre-test, post-test) multivariate repeated measures analysis of variance (ANOVA). We explored the effects of the physical activity intervention on aerobic fitness (VO_2max_), and estimates of white matter microstructure (FA) in pre-specified ROIs. If the interaction was significant, we conducted paired *t*-tests and independent *t*-tests to further explore group differences in white matter structural changes from pre-test to post-test. For those tracts showing significant changes in FA (*p* < 0.05), we conducted secondary analyses on RD and AD to better understand the underlying biological properties of overall FA changes in white matter microstructure.

## Results

### Participant Demographics and Aerobic Fitness

Group demographic and aerobic fitness data at pre-test and post-test are provided in Table 1. The variables of age, sex, race, IQ, SES, pubertal timing, VO_2_max, and BMI did not differ between the physical activity and control groups (all t < 1.0, p > 0.05).

**Table 1:** Mean (*SD*) for physical activity and wait list control groups at pre-test and post-test.

	**Physical activity**		**Control**	
	**Pre-test**	**Post-test**	**Pre-test**	**Post-test**
Age (years)	8.7 (0.57)	9.5 (0.61)	8.7 (0.5)	9.4 (0.51)
Gender	39 girls, 37 boys		34 girls, 33 boys	
IQ	110.4 (16.9)		111.7 (12.0)	
Pubertal timing	1.39 (0.47)		1.37 (0.45)	
SES	1.91 (0.79)		1.90 (0.76)	
VO_2__max_ (mL/kg/min)	42.26 (7.9)	42.86 (7.8)	42.96 (6.9)	42.95 (6.1)
VO_2__max_ percentile	34.80 (31.2)	36.33 (31.1)	38.69 (29.4)	37.1 (26.9)
BMI (kg/cm^2^)	19.0 (3.7)	19.2 (3.68)	18.7 (4.06)	19.4 (4.52)
FA genu of corpus callosum	0.750 (0.022)*	0.755 (0.021)*	0.754 (0.021)	0.754 (0.022)
RD Genu (mm^2^/s)	0.00032 (0.00003)*	0.00030 (0.00004)*	0.00031 (0.00003)	0.00031 (0.00003)
AD Genu (mm^2^/s)	0.00148 (0.00006)	0.00148 (0.00005)	0.00149 (0.00005)	0.00149 (0.00007)
FA body of corpus callosum	0.694 (0.034)	0.703 (0.025)	0.696 (0.034)	0.700 (0.025)
FA splenium of corpus callosum	0.789 (0.022)	0.797 (0.022)	0.795 (0.022)	0.798 (0.014)
FA corona radiata	0.506 (0.023)	0.514 (0.030)	0.505 (0.023)	0.509 (0.022)
FA superior longitudinal fasciculus	0.514 (0.028)	0.520 (0.033)	0.517 (0.025)	0.520 (0.025)
FA posterior thalamic radiation	0.618 (0.032)	0.622 (0.029)	0.623 (0.032)	0.622 (0.030)
FA uncinate fasciculus	0.533 (0.056)	0.540 (0.042)	0.549 (0.052)	0.541 (0.045)

IQ, woodcock johnson III paper and pencil task of general intellectual ability (Woodcock, 1997); SES, socioeconomic status (Low: < 2; Moderate: 2-3; High, >3). ^*^p < 0.05.

There were no significant effects of the intervention on aerobic fitness (p>0.05), indicating that the physical activity dose provided in the intervention did not significantly modulate aerobic fitness levels. Descriptively, the physical activity intervention group exhibited a non-significant gain in VO_2max_ percentile of 1.53% as a function of their daily exposure to physical activity, compared to a 1.62% decrease in VO_2max_ percentile in the wait-list control group over the same period.

### White Matter Microstructure

**Figure 2:**
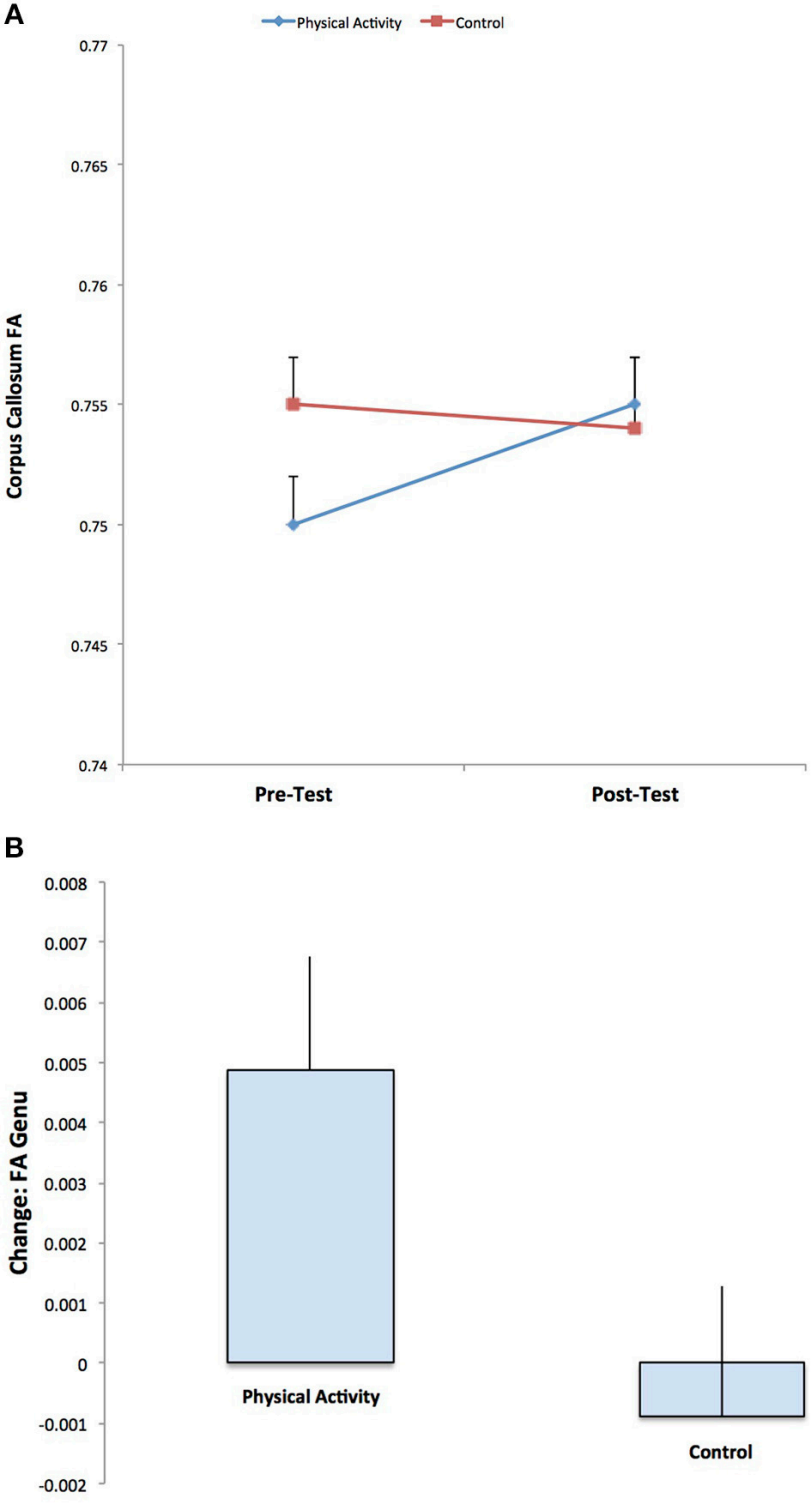
Illustration of the significant Group × Time interaction for FA in the genu of the corpus callosum, with the physical activity group showing significant increases in FA from pre-test to post-test, and no changes in FA for the control group. The result suggests increased estimates of white matter microstructure and fiber integrity with physical activity participation.

DTI estimates of white matter microstructure at pre-test and post-test are provided in Table 1. Consistent with our hypotheses, there was a significant Group × Time interaction for FA in the genu of the corpus callosum (*F*_(1, 141)_ = 3.973, *p* = 0.048), with the physical activity group showing significant increases in FA from pre-test to post-test (*t*_(75)_ = 2.551, *p* = 0.013), and no changes in FA for the control group (*p* = 0.686) (Figure 2). There were no group differences in FA at pre-test or post-test.

**Figure 3:**
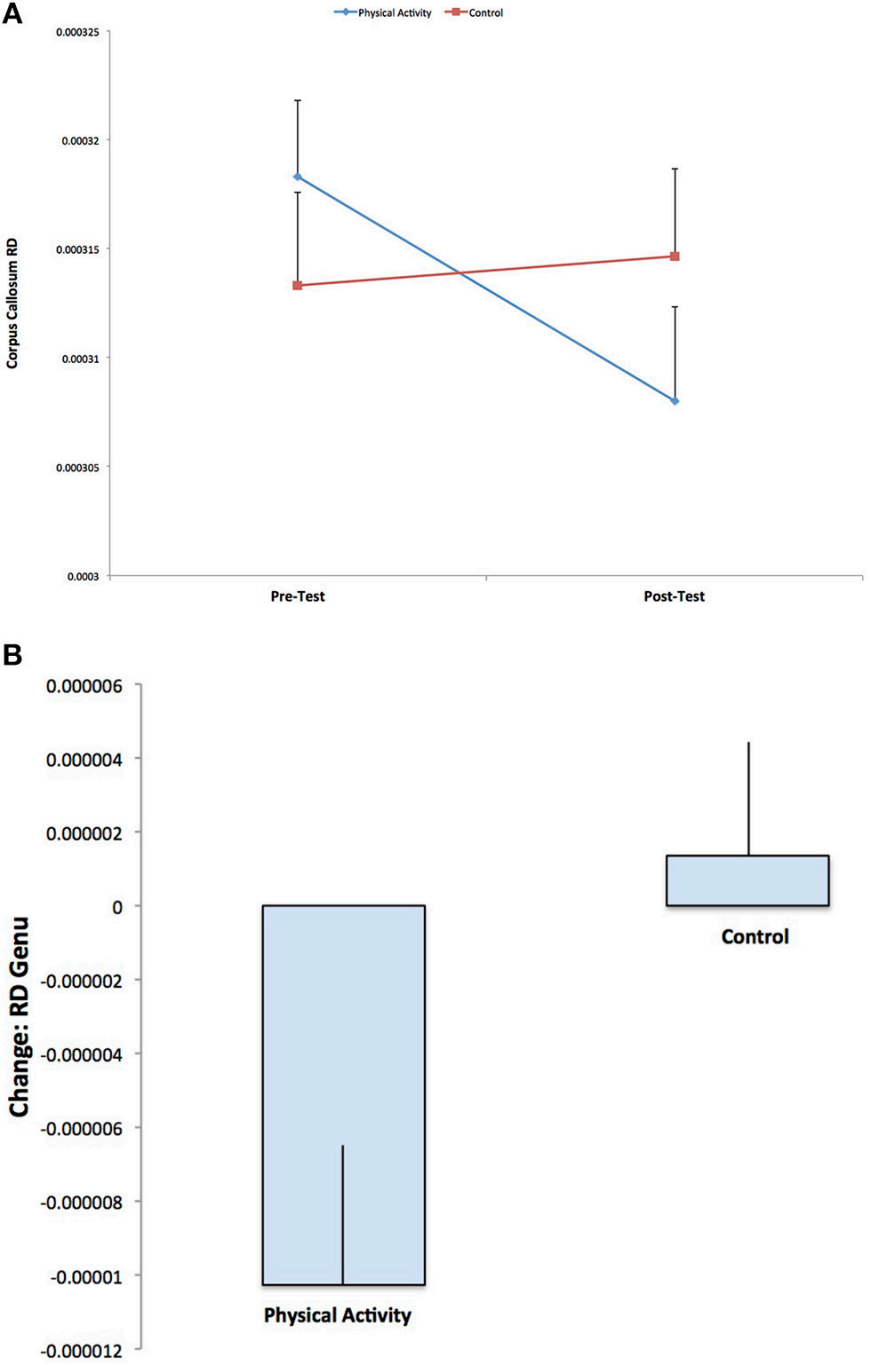
Illustration of the significant Group × Time interaction for RD in the genu of the corpus callosum, with the physical activity group showing significant decreases in RD from pre-test to post-test, with no changes in RD for the control group (*p* = 0.592). The result suggests increased estimates of white matter myelination with physical activity participation.

A secondary analysis showed a Group × Time interaction for RD in the genu of the corpus callosum (*F*_(1, 141)_ = 5.467, *p* = 0.021), with the physical activity group showing significant decreases in RD from pre-test to post-test (*t*_(75)_ = 2.705, *p* = 0.008), with no changes in RD for the control group (*p* = 0.663) (Figure 3). There were no group differences in RD at pre-test or post-test. There were no effects for AD in the genu of the corpus callosum (*p* > 0.05).

No Group × Time interactions reached significance for FA of the corona radiata, superior longitudinal fasciculus, posterior thalamic radiation, or uncinate fasciculus.

## Discussion

The present study demonstrates that 7- to 9-year-old children who participated in an after-school physical activity program showed increased white matter microstructure in the genu of the corpus callosum, with no changes in white matter microstructure in the wait list control group. The results were specific to the anterior corpus callosum, with physical activity participation leading to greater increases in FA, greater decreases in RD, and no changes in AD from pre- to post-test. There were no group differences in white matter microstructure at pre-test or post-test. The findings suggest that physical activity may lead to more tightly bundled and structurally compact fibers (FA) and increased myelination (RD), with no changes in estimates of axonal fiber diameter (AD). We did not demonstrate effects of physical activity participation on the structure of the corona radiata, superior longitudinal fasciculus, posterior thalamic radiation, or uncinate fasciculus. In general, these results demonstrate that 7- to 9-year-old children who participate in moderate to vigorous physical activity, 5 days per week, for 9 months, experience changes in the microstructural properties of the anterior corpus callosum. This effect was not realized in a wait list control group that was not involved in an after-school physical activity program and reflects typical development.

Our results have broad implications. The corpus callosum integrates cognitive, motor, and sensory information between the left and right hemispheres of the brain, and the white matter tract plays a role in cognition and behavior (Banich and Brown, 2000). As this tract is undergoing developmental changes during pre-adolescence, our results raise the possibility that incorporating physical activity into a child's day may enhance the development of these white matter fibers. Moreover, the corpus callosum is associated with a broad array of clinical syndromes. Abnormal structural development of the corpus callosum has been found to relate to cognitive and behavioral deficits in children with neurodevelopmental disorders including attention-deficit hyperactivity disorder, autism, and schizophrenia (Swayze et al., 1990; Hynd et al., 1991; Barnea-Goraly et al., 2004). Thus, the corpus callosum plays a key role in efficient transmission of information between brain hemispheres to support cognitive function.

In fact, physical activity may capitalize on the structure of white matter fibers in the anterior corpus callosum. The specificity of our physical activity results to the corpus callosum are consistent with the effects of a 6-month aerobic exercise intervention in older adults, in which physically active older adults showed greater increases in white matter volume of the anterior corpus callosum relative to a stretching and toning control group (Colcombe et al., 2006). Note that a volumetric approach to assess white matter (Colcombe et al., 2006) differs from DTI techniques, which explore microstructural properties of brain tissue. In addition, a case study of Olga Kotelko, a world-famous non-agenarian track-and-field athlete with over 30 world records in her age category (90–95 years), only showed higher FA in the genu of the corpus callosum compared to a reference sample of low active women (age 60–78 years) (Burzynska et al., 2015). The present results also address the larger debate about the capability for plasticity in white matter structure (Sexton et al., 2016). To date, there is scant evidence that interventions can reliably alter white matter structure (Sexton et al., 2016). But, the results we describe here provide evidence that a physical activity intervention is capable of modifying white matter structure in children.

The analysis of different diffusion properties (FA, RD, AD) allowed us to speculate about the specific effects of physical activity on changes in properties and biological mechanisms of white matter microstructure in preadolescent children. Our results suggest that physical activity during childhood may influence fiber structural integrity and fiber alignment (FA) in tracts, perhaps via increased myelination (RD), and distinct from axonal properties (AD) (Song et al., 2003, 2005; Sun et al., 2006, 2008). Indeed, aerobic exercise leads to many molecular and cellular changes in the brain that may influence white matter. For example, exercise has been found to improve cerebrovascular health and cerebral perfusion, which may benefit white matter via improved oxygen and nutrient delivery (Black et al., 1990; McDonnell et al., 2013; Chaddock-Heyman et al., 2016). In non-human animal models, exercise benefits the vascular system via increased capillary density (Black et al., 1990; Isaacs et al., 1992; Neeper et al., 1995; Carro et al., 2001; Swain et al., 2003; Ding et al., 2006; Clark et al., 2009). In younger and older humans, regular aerobic exercise has been associated with a healthier vascular profile involving blood pressure, vascular resistance, and arterial elasticity (McDonnell et al., 2013). In children, higher levels of aerobic fitness have been found to relate to greater perfusion in the hippocampus, suggesting improved microcirculation and cerebral vasculature (Chaddock-Heyman et al., 2016). Thus, it is possible that benefits to the vascular system, via improved oxygen and nutrient delivery, mediate some of the effects of physical activity on white matter structure during childhood.

Aerobic exercise also leads to increased production of growth factors. In particular, brain-derived neurotrophic factor (BDNF) is known to play a role in neuron growth and survival, synaptic plasticity, and axonal pruning and regeneration (Mamounas et al., 2000; Cao et al., 2007; Singh et al., 2008). In older adults, increased BDNF expression has been associated with physical activity-related changes in hippocampal volume and improved functional connectivity (Voss et al., 2010; Erickson et al., 2011). Exercise also upregulates IGF-1, a neurotrophic factor involved in proliferation of oligodendrocytes which help allow for axonal myelination (Krityakiarana et al., 2010; Matsumoto et al., 2011). Thus, an upregulation of neurotrophic and growth factors with participation in physical activity may also play a role in changes in white matter microstructure in children.

Our results also raise the possibility that physical activity and aerobic fitness may relate differently to white matter health. We do not demonstrate effects of the physical activity intervention in the same tracts that differ in microstructure in higher fit (>70th percentile VO_2max_) and lower fit (< 30th percentile VO_2max_) children (Chaddock-Heyman et al., 2014a). It is possible that different biological mechanisms drive associations between physical activity and white matter structure, vs. aerobic fitness and white matter structure. Our cross-sectional comparison of higher fit and lower fit children (Chaddock-Heyman et al., 2014a) compared estimates of white matter microstructure in extreme aerobic fitness groups, whereas the present randomized controlled trial specifically enrolled lower fit participants that remained lower fit even after the completion of the intervention (i.e., average VO2max percentile of 36% at pre-test and post-test, across groups). In addition, the children in the physical activity program only showed a 1.5% increase in VO2max, thereby moving from a lower fit classification to a slightly less lower fit classification. Hence, if a threshold for aerobic fitness is necessary to engender differences in white matter structure, it was likely not achieved with the daily dose of physical activity administered via the FITKids2 program. This result suggests that changes in aerobic fitness might not be the primary mediator for the physical activity-related changes in white after microstructure. Finally, as our results do not replicate the effects of physical activity on white matter structure in the superior longitudinal fasciculus or uncinate fasciculus in a small sample of overweight children (Krafft et al., 2014; Schaeffer et al., 2014), future research may explore the role of interactions among physical activity, aerobic fitness, and adiposity in brain development during childhood.

The present study provides an additional step in identifying the relationship between physical activity and white matter microstructure during typical child development, but we make conclusions in the context of the limitations to our study. The use of a wait list control group makes it difficult to conclude that our reported group differences are entirely based on daily physical activity participation; however, others (Krafft et al., 2014) have shown DTI findings following a physical activity intervention relative to an active control group. Here we interpret the wait list control group as a group of typically developing children across a period of 9 months. Furthermore, it is possible that other aspects of our multimodal after-school program, which included aerobic, motor, and social activities as well as a brief educational component, may have contributed to the results, such that an enriched after-school program benefits brain health in children. Finally, it is important for future research to link the reported associations between physical activity and white matter structure to cognition and scholastic performance. It is possible that changes in structural connectivity with physical activity may account for some differences in cognitive performance. In fact, the corpus callosum has been found to play a role in attention, memory, and processing speed across the lifespan (Madden et al., 2012). Also, we note that because DTI does not measure tissue parameters (e.g., fiber integrity, myelination) directly, but rather, measures the displacement of water molecules, the underlying microstructural properties of white matter can only be inferred from this displacement.

In conclusion, a 9-month randomized controlled physical activity intervention significantly improved estimates of structural integrity and myelination of the genu of the corpus callosum. Given that no significant changes were observed for children assigned to the wait list control group, the key implication from this study is that participating in an after-school physical activity program enhances the microstructure of the anterior corpus callosum, which may suggest faster neural conduction between brain hemispheres. These results arrive at an important time, as children become increasingly unfit and sedentary, and educators reduce or eliminate opportunities for physical activity during the school day in favor of academic topics (Centers for Disease Control and Prevention, 2010). Hopefully these findings will reinforce the importance of physical activity during development and drive public health change in promoting physical activity opportunities for children.

## Author Contributions

LC-H, DC, CH, and AK conceived and designed the study. LC-H analyzed the main outcomes of the present manuscript and wrote the paper. LC-H, CK, ED, LR, S-CK, JB, and RW involved in subject running, data analysis, and data organization of FITKids2 data. KE, CH, and AK Paper feedback.

### Conflict of Interest Statement

The authors declare that the research was conducted in the absence of any commercial or financial relationships that could be construed as a potential conflict of interest.
